# Insulin/IGF-I and Related Signaling Pathways Regulate Aging in Nondividing Cells: from Yeast to the Mammalian Brain

**DOI:** 10.1100/tsw.2010.8

**Published:** 2010-01-21

**Authors:** Edoardo Parrella, Valter D. Longo

**Affiliations:** Division of Neurogerontology, Andrus Gerontology Center and Department of Biological Sciences, University of Southern California, Los Angeles, CA, USA

**Keywords:** aging, IGF-I, TOR, SIRT1, oxidative damage, neurons, cognitive function, brain, yeast, nematode, fly

## Abstract

Mutations that reduce glucose or insulin/insulin-like growth factor-I (IGF-I) signaling increase longevity in organisms ranging from yeast to mammals. Over the past 10 years, several studies confirmed this conserved molecular strategy of longevity regulation, and many more have been added to the complex mosaic that links stress resistance and aging. In this review, we will analyze the similarities that have emerged over the last decade between longevity regulatory pathways in organisms ranging from yeast, nematodes, and fruit flies to mice. We will focus on the role of yeast signal transduction proteins Ras, Tor, Sch9, Sir2, their homologs in higher organisms, and their association to oxidative stress and protective systems. We will discuss how the “molecular strategy” responsible for life span extension in response to dietary and genetic manipulations appears to be remarkably conserved in various organisms and cells, including neuronal cells in different organisms. Taken together, these studies indicate that simple model systems will contribute to our comprehension of aging of the mammalian nervous system and will stimulate novel neurotherapeutic strategies in humans.

## INTRODUCTION

Aging is an extremely complex process that has stimulated many theories to explain it. Among the different putative mediators of aging, oxidative damage is perhaps the most widely accepted and among the oldest proposed[1]. Oxidative damage to macromolecules is believed to be caused by an imbalance between reactive oxygen species (ROS) and antioxidant systems, which include enzymes, such as the superoxide dismutases (SODs), catalase and glutathione peroxidase, and small molecules such as vitamins C and E. Aging is accompanied by progressive accumulation of oxidative damage in many tissues, including the peripheral and central nervous systems (PNS, CNS)[2,3,4,5]. The brain, in particular, is highly vulnerable to oxidative damage because of its high oxygen consumption (20% of the oxygen consumed by the whole body) and an antioxidant system that is not capable of preventing oxidative damage accumulation at advanced ages[6]. Age-dependent oxidative damage can lead to behavioral deficits in rodents[7] and possibly in humans. In fact, several studies have described an increased level of oxidative damage to nucleic acid, proteins, and lipids in the older human brain[8,9,10]. Oxidative damage is believed to play a major role in a variety of neurodegenerative diseases, including Alzheimer's disease (AD)[11], Parkinson's disease (PD)[12], and Huntington's disease (HD)[13], although it is not clear whether oxidative stress is the primary cause of any of these diseases, or simply the consequence of damage to systems that produce and scavenge ROS caused by other sources of cellular damage, such as, for example, the presenilin mutations that lead to early-onset AD[14,15].

Biogerontology studies performed in the past decade indicate that dietary changes or genetic manipulations extend longevity by producing a shift to a maintenance mode characterized by enhanced stress resistance. The longevity and stress-resistance regulation can be obtained by specific mutations that decrease the activity of insulin/insulin-like growth factor-I (IGF-I) signaling pathways or by reducing calorie intake by at least 30%, with a strategy called dietary or calorie restriction (CR)[16,17]. The described regulation appears to have evolved to promote longevity during periods of starvation and is effective in a wide range of organisms.

## YEAST

### Yeast: Chronological Life Span vs. Replicative Life Span

Chronological life span (CLS) in yeast is based on the days of survival of populations of nondividing cells[18]. This paradigm is obtained by growing and maintaining yeast cells in minimal glucose medium, or by transferring the organisms to water (starvation or extreme CR) after 72 h. After the growth phase, characterized by fermentative catabolism of glucose, the yeast population shifts to a largely respiratory metabolism. The viability of the aging yeast is assessed over time[19].

Aging in yeast is also measured by monitoring the number of daughter cells generated by an individual mother cell (replicative or budding life span, RLS). Since daughter cells are smaller and easily recognizable, they can be removed by micromanipulation when budding occurs.

CLS, measured as long-term survival of cells maintained in stationary phase, represents a valuable paradigm to study stress resistance and longevity in nondividing cells. Oxidative damage in growing cells is reduced over time and diluted by the synthesis of new macromolecules required for rapid growth. For example, yeast cells can grow in the presence of elevated concentrations of superoxide and $H_2O_2$[20,21]. In contrast, nondividing yeast obtain most of their cellular energy from mitochondrial respiration, as is observed in postmitotic cells of several mammalian tissues. While in this stationary and oxidative phosphorylation-based phase, yeast shows several cellular alterations caused, in part, by the accumulation of oxidative damage, which are also observed in mammalian cells from old organisms, including increased DNA damage and defects in re-entering the growth phase. As mitochondrial-derived ROS are believed to contribute to the aging of postmitotic cells, such as neurons, the yeast CLS system provides a particularly simple, but valuable, model system to understand the fundamental aspects of aging in postmitotic mammalian cells.

### Regulation of Longevity and Stress Resistance in Yeast

Over the past 10 years, many yeast genes that regulate longevity and stress resistance have been identified. Remarkably, these genes are part of glucose signaling pathways and are related to mammalian genes that are activated by the presence of nutrients and growth factors. Several studies suggest that down-regulation of nutrient signaling pathways can extend yeast longevity by activating stress-resistance transcription factors that regulate the expression of genes involved in protection against oxidative and heat stress, DNA repair, and metabolism. Moreover, these signaling pathways have been implicated in regulating CR-induced life span extension[22].

The two major nutrient signaling pathways implicated in the yeast life span regulation are the Tor/Sch9 and the Ras/AC/PKA pathways (see Fig. 1). Deletion of the signal-transduction proteins RAS2, TOR, CYR1 (adenylate cyclase, AC), and SCH9 increases life span as well as oxidative stress resistance and thermotolerance. Interestingly, the beneficial effects of CR are reduced or blocked in mutants that are missing these proteins, indicating that their down-regulation mediates part of the CR-dependent effects on longevity (see Fig. 1).

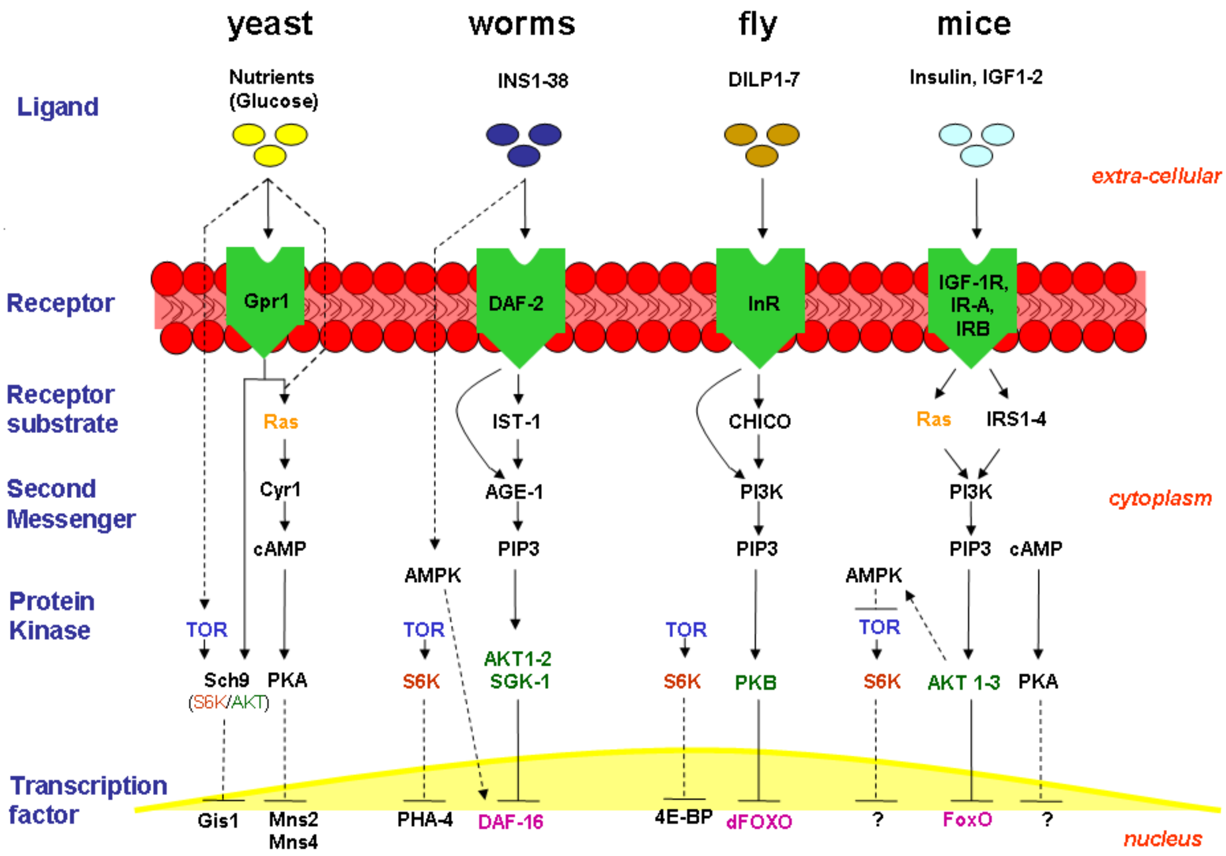

**FIGURE 1**. A simplified depiction of the glucose and insulin/IGF-I pathways in yeast, worms, flies, and mice. The signaling pathway starts when the ligands bind the appropriate receptor. For yeast, the ligand is represented mainly by glucose and amino acids. Worms have 38 ligands discovered so far ($INS_{1-38}$), flies seven ($DILPs_{1-7}$), mice three (insulin, IGF-I, and IGF-II). The yeast receptor is mainly represented by Gpr1 (for a complete review, see Gancedo[184]). Worms and flies present only one receptor (DAF-2 and InR, respectively), whereas mice present three (IGF-IR, IR-A, IR-B, and other related growth factor receptors). The signal is then transduced from the receptor via adaptor proteins: the GTPase Ras in yeast, the worm IST-1, and the fly CHICO, the respective homologs to vertebrate insulin receptor substrates, the four insulin receptor substrates ($IRS_{1-4}$) and also Ras in mice. In worms and flies, the signal can be also transduced directly to PI3K (AGE-1 in worms). PI3K converts $PIP_2$ into the second messenger $PIP_3$ in worms, flies, and mice proaging pathways. Cyr1 (AC) instead catalyzes the conversion of ATP to generate the second messenger cAMP in yeast. Elevated levels of the second messengers activate Ser/Thr-kinases: Sch9 and PKA (homologs of the protein kinase Akt and S6K) in yeast; Akt-1, Akt-2, SGK-1 (serum- and glucocorticoid-induced protein kinase-1) in worms; PKB in flies; Akt-1, Akt-2, and Akt-3 in mice. Yeast Sch9 has been shown to function directly downstream of the TOR kinase protein[23]. TOR and S6 kinase have also been implicated in worms, flies, and mice longevity pathways. Activated kinases phosphorylate specific transcription factors, causing their inactivation: the stress-resistance transcription factors Mns2, Mns4, and Gis1 in yeast and forkhead transcription factors, and other transcription factors in higher eukaryotes. Worms and flies contain a single forkhead transcription factor (Daf-16 and FOXO, respectively); mice display four members of the FoxO family called FoxO-1, -3, -4, and -6 (for a review, see Birkenkamp and Coffer[185]). This list focuses on the genes and pathways that play partially conserved aging regulatory roles from yeast to mammals. Many other genes not shown here have been implicated in life span regulation.

SCH9, an AGC serine/threonine kinase that shares high sequence identity with the mammalian kinases Akt/PKB and S6K (S6 kinase), but that is more functionally related to S6K[23,24], is part of a nutrient-sensing pathway whose down-regulation increases the CLS by up to threefold[25]. More recent papers indicate that Sch9 inactivation also extends the yeast RLS[26,27]. Sch9 has been shown to function directly downstream of the TOR (Target of Rapamycin) kinase protein[23]

Two TOR orthologs, *TOR1* and *TOR2*, have been discovered in yeast. Both Tor1 and Tor2 mediate growth-related signaling in a rapamycin-sensitive manner. Reduction of the TOR complex I (TORC1) activity results in an extension of yeast RLS similarly to what has been observed with Sch9 inactivation[26,27]. Moreover, recent papers support a role for the down-regulation of the TOR pathway in CLS extension[28,29], confirming the existence of a Tor/S6 kinase life span regulatory pathway in yeast.

The Ras G-proteins are also evolutionarily conserved, and involved in monitoring the nutritional status of the cell and regulation of the cell cycle[30]. The deletion of *RAS2* doubles the CLS of yeast by activation of stress-resistance transcription factors Mns2/Mns4 and SODs, through the down-regulation of AC and PKA[19,25].

The essential function of antioxidant protection in longevity is also supported by the shorter life span of mutants lacking either SOD1 (CuZnSOD, cytoplasmic superoxide dismutase) or SOD2 (MnSOD, mitochondrial superoxide dismutase) compared to wild type[20], or the inactivation of aconitase. Aconitase, which is a citric acid cycle 4Fe-4S cluster enzyme, is very sensitive to superoxide and is inactivated during aging in wild-type cells, but much less in long-lived mutants[19,25,31].

In fact, the overexpression of both SOD1 and SOD2 reduces aconitase inactivation and increases life span by 30%, while overexpression of each SOD alone causes a small, but significant, survival extension[19,25]. Catalase may also protect against aging, but the effect of either its deletion or overexpression on life span is much smaller[32]. Furthermore, recent studies have demonstrated that longevity extension in mutants lacking *TOR1* is accompanied by increased SOD2 expression, but can still be achieved in the absence of SOD2 activity[28]. These results do not suggest that SOD2 is not important for delaying aging since other systems activated in *tor1* mutants could compensate for the lack of SODs.

Notably, overexpression of multiple antioxidant enzymes can extend longevity by up to 30%, whereas multiple mutations in glucose signaling pathways can cause a fivefold extension in survival, indicating that increased antioxidant protection is only one of the many components of a program aimed at reducing growth and promoting protection and longevity.

For example, Sch9 as well as Tor and Ras2 regulate multiple metabolic pathways, such as that controlling a shift from an ethanol generation and catabolism mode to a glycerol production mode[33]. As for antioxidant protection, this carbon source substitution contributes to the delay of aging by generating an environment that mimics CR.

Another fundamental key modulator of stress response and life span in yeast and higher eukaryotes is SIR2 (for reviews see Longo[34] and Longo and Kennedy[35]). SIR2 is a conserved deacetylase that requires NAD as cofactor[36].

Several studies have proposed a role for SIR2 in increasing yeast RLS[37] and in mediating the effects of CR, although the link between Sir2, CR, and RLS is controversial[38,39]. Our findings suggest that SIR2 can have the opposite effect on CLS. In fact, deletion of SIR2 does not cause any significant effect on the CLS of wild-type yeast cells, but the lack of Sir2 coupled with severe CR or mutations in the Ras or Sch9 pathways can cause an additional extension of CLS[40]. By contrast, Sir2 overexpression reduces the life span extension promoted by lack of SCH9[40].

## WORMS

There are remarkable similarities between the genes involved in longevity extension in *Saccharomyces cerevisiae* and *Caenorhabditis elegans* (see Fig. 1). Mutations in the *C. elegans* daf-2 gene (abnormal Dauer Formation-2), encoding the worm insulin/IGF-I receptor, increase life span by down-regulating age-1 (AGEing alteration-1), the gene encoding the catalytic subunit of the worm PI3K (phosphoinositide

3-kinase)[41,42,43,44], and the downstream AKT, which results in the activation of the forkhead transcription factor DAF-16[45,46,47].

Yeast Sch9 is 47 and 45% identical to worm AKT-2 and AKT-1, respectively, and functions downstream of the insulin homolog DAF-2[48,49]. Sch9 is functionally more closely related to S6K, which may also be linked to the insulin/IGF-I–like signaling pathway in worms and higher eukaryotes[50]. Parallel to findings on yeast, among the genes regulated by daf-2 through the forkhead transcription factor DAF-16, are SOD2 and catalase[51,52] heat shock proteins[53], and genes involved in drug detoxification[54]. SOD/catalase mimetics protect against oxidative stress and increase life span in *C. elegans*[55]. Moreover, similarly to the Ras pathway, daf-2 down-regulates storage of reserve nutrients as fat and glycogen and the switch to the hypometabolic dauer larva state[41,42,47].

TOR deficiency has been shown to influence life span in nematodes. Mutations or inhibition of let-363 or daf-15, the respective homologs of TOR kinase and RAPTOR (a TOR-interacting protein), enhance life span up to twofold[56,57]. Also, CR increases life span in nematodes, and TOR and sir2.1 are implicated in this action (for a review, see Houthoofd and Vanfleteren[58]). Recent studies show that glucose deprivation can extend life span in worms, whereas glucose availability decreases *C. elegans* life span[59]. Interestingly, Sir2.1 signaling is not required for life span extension under glucose restriction, since deficiency of glucose can also increase life span in different worm strains lacking Sir2.1[59].

Mutations that lower insulin pathway signaling also increase innate immunity[60], and delay age-dependent impairment of pharyngeal pumping and body movements, two markers of senescence in nematodes[61].

It has been shown that the age-1/daf-2 insulin-like pathway plays a specific role in neurons to regulate longevity[62,63]. Although structurally simple, the *C. elegans* nervous system provides the basic circuit that underlies the association between external stimuli and behavioral output. Despite the fact that *C. elegans* neurons maintain their structural integrity during aging, old nematodes present age-dependent behavioral decline[64]. Instead, long-lived daf-2 and age-1 mutants also exhibit good performance in thermotaxis learning tests in old age[65]. Since changes in locomotor activity do not seem to be involved, it is likely that the described life-extending mutations can provide neuroprotection against the age-dependent impairment in associative learning behaviors. Finally, many mutants in chemosensory cilia, specific neurons whose function is to sense environmental chemical signals, exhibit extended life span, dependent, at least in part, on DAF-16 activity[66]. Some of the long-lived mutants also display resistance to the oxidative stressor paraquat, although the oxidative stress resistance does not appear to be correlated to DAF-16[67].

## FLIES

A number of the same genes implicated in life span regulation in yeast and worms are associated with aging in the fruit fly *Drosophila melanogaster*. For example, mutations in dINR and chico, the fruit fly insulin receptor and insulin receptor substrate, respectively (see Fig. 1), extend life span[68,69] and also delay cardiac aging[70]. Moreover, mutations in dINR can also cause an increase in storage of nutrients and SOD expression[69], and increased resistance to exogenous oxidative stress[69]. As observed in yeast and worms, inhibition of TOR signaling by overexpression of Tsc1 and Tsc2 (tuberous sclerosis complex 1 and 2), or by knocking down TOR or S6K, causes life span extension in fruit flies[71].

In *Drosophila*, the fly ortholog dSir has been reported to increase life span as well[72].

Also in flies, dietary restriction can extend life span (for a review, see Tatar[73] and Piper and Partridge[74]). This effect can be caused, at least in part, by down-regulation of the TOR pathway since life span of mutant flies with low TOR activity is only modestly extended under nutrient-limitation conditions[71].

Oxidative stress and antioxidant enzymes also play a crucial role in *Drosophila*. Several studies support the existence of an inverse correlation between oxidative damage to proteins and DNA, and expectancy of life in fruit flies[75,76]. Overexpression of SOD1 and of mitochondrial SOD2[77] in fruit flies increase both mean and maximum life span[78,79]. Similar effects have been observed by administering exogenous antioxidants[80]. Furthermore, similarly to the observations in yeast, activity of the mitochondrial enzyme aconitase is significantly decreased in old flies[81], and the systemic knockdown of Sod1 decreases life span and accelerates age-related locomotor impairment[82]. Short-lived flies lacking SOD2 show an accelerated decay of several functional measures of aging, including cardiac and locomotor activities and olfactory behavior[83,84]. However, in other studies, overexpression of SOD1 does not cause any effect on life span[85,86] and it also has been suggested that overexpression of antioxidant proteins only increases life span in short-lived *Drosophila* backgrounds[87].

The *Drosophila* CNS shows markers of aging that indicate that it can serve as a simple system to study brain aging and diseases in humans. Old flies display impairment in behavioral tests including the Pavlovian olfactory association[88] and reproductive behavior assays[89]. In the last example, the impairment appears to be directly related to age-dependent degeneration of dopaminergic neurons[89]. Studies based on negative geotaxis as an index of locomotor function, a measure of neurodegeneration and age-related functional decline, suggested that the chico mutation can attenuate age-related locomotor impairment in flies[90]. Recently, to support these findings, a forward genetic screen in *Drosophila* indicated a delay in age-related locomotor impairment with loss of function alleles implicated in insulin signaling, including Akt, Dp110 (respectively, the genes for AKT and the catalytic subunit of PI3K), and the gene phosphoinositide-dependent kinase 1 (PDK1)[91]. Interestingly, only some of the fly strains that exhibited age-related locomotor impairment also showed strengthened resistance to the oxidative stressor paraquat. These findings suggests that in *Drosophila*, enhanced resistance to oxidative stress (at least for paraquat) is not required for the locomotor function protection in the elderly.

Glia cells also play an important role in aging and neuroprotection in the fruit fly. In fact, loss of the Glial Lazarillo (GLaz) gene, a homolog of vertebrate apolipoprotein D (ApoD), mainly expressed in the fruit fly glia cells, shortens life span, reduces insect resistance to oxidative stress, and accelerates motor systems senescence[92,93].

## MAMMALIAN CELLS AND RODENTS

### Insulin/IGF-I and Connected Signaling Pathways in Mammals

The homologs of proaging genes in lower organisms described in the previous sections, including IGF-IR, PI3K, Ras, Akt, Tor, and S6K, are important growth and survival mediators of IGF-I signaling in mammalian cells[16] (see Fig. 1).

These genes and some of the pathways in which they function appear to carry out key functions that promote sensitivity to stress and aging in mammals, although they can also promote protection by blocking the activation of apoptotic pathways. For example, several mouse models that carry mutations in the growth hormone (GH)/insulin/IGF-I axis have been generated and were shown to extend survival, as reviewed recently by Kopchick and colleagues[94]. Examples include mice that lack GH and other pituitary hormones (Snell and Ames dwarf mice)[95,96], mice with mutations in GHRHR (growth hormone releasing hormone receptor) (Lit/Lit mice)[97], mice lacking GH receptor/binding protein genes (Laron mouse)[98], and heterozygous knockout mice for IGF-I receptor[99]. The mutated mice listed above or cells derived from several of them are characterized by an improved resistance to stress. Whereas deficiencies in either GH or GHR have consistently been associated with life span extension of 30% or higher, studies of the effects of IGF-IR and of downstream proteins on life span are not conclusive. For example, only female IGF-IR$^{+/-}$ mice are long lived and this longevity was not confirmed in a different genetic background[100]. Results correlating life span and IRS (the receptors substrates of insulin/IGF-I signaling, see Fig. 1) in mouse are also contradictory. Recent longevity studies have shown that female IRS-1$^{-/-}$ mice exhibit significant longevity extension compared to controls[101], and mice with conditional disruption of the IRS-2 gene in the brain (bIRS-2$^{-/-}$) display significant extension in life span[102]. However, other studies showed that a reduction of GH or IGF-I levels does not always correlate with improvement in life span. In fact, mice that express a transgene for growth hormone antagonist (GHA), even though exhibiting decreased levels of IGF-I, have no extension in life span when compared to sibling controls[103], although it is possible that the optimal reduction in IGF-I was not achieved in these mice.

CR is well known also to extend life span in mammals. Survival extension promoted by CR involves complex and poorly understood mechanisms that appear to overlap partially with those responsible for the effects of insulin/IGF-I signaling on aging[36,104] (see Figs. 1 and 2). Many phenotypic characteristics identified in calorie restricted animals, including reduced body weight, reduced IGF-I levels, insulin sensitivity, and resistance to certain diseases, are shared with mice with reduced GH/GHR activity. However, comparisons of gene expression patterns in mice subjected to CR and mutants for insulin/IGF-I pathway have revealed numerous differences[94], confirming that the overlap between the pathways altered by these nutritional and genetic interventions is only partial.

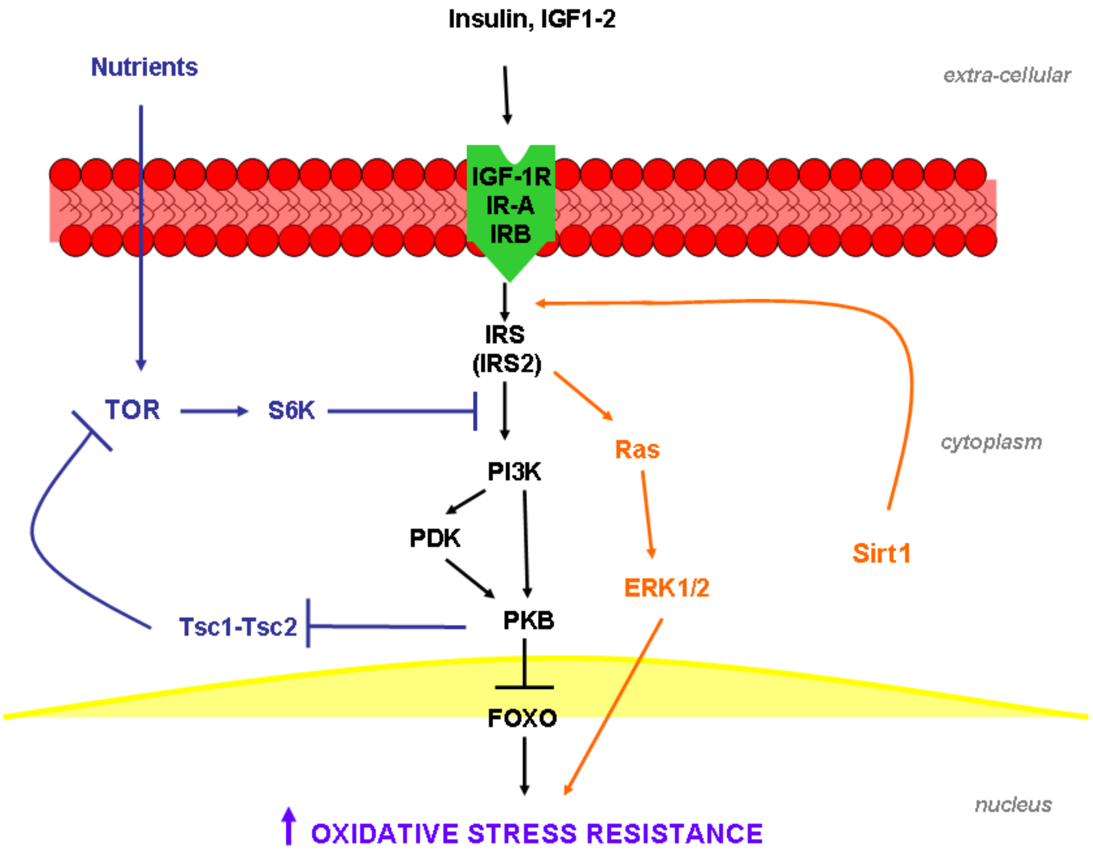

**FIGURE 2.** Interactions between insulin/IGF-I, TOR, and SIRT1 pathways in mammals. Nutrients, mainly amino acids, promote the activity of the TOR kinase. TOR, in its turn, activates S6K, a crucial AGC kinase involved cell growth, which can inhibit PI3K activation. Activated PKB activates TOR signaling via inhibition of TSC-1 and -2. The SIRT1 interactions represented in the figure have been observed in neurons[163,172]. SIRT1 promotes activation of the IRS-2 and Ras/ERK1/2 pathway, suggesting that SIRT1 may strengthen IGF-I signaling, in part, by deacetylating IRS-2. Inhibition of SIRT1 reverts the described mechanisms and enhances resistance to oxidative stress.

As observed in lower eukaryotes, there is also a strong inverse association between insulin/IGF signaling and oxidative stress in mammals. For example, antioxidant enzymes, such as catalases and SODs, are reduced in cells obtained from mice that overexpress GH, or treated with GH or IGF-I[105,106]. Although overexpression of antioxidant enzymes, such as SODs, increases life span and stress resistance, while the down-regulation of those enzymes produces opposite effects, the correlation between aging and SODs is complex. In mice, the deletion of SOD2 is lethal, causing massive oxidative damage and death shortly after birth[107,108]. Mice heterozygous for a null mutation of SOD2 ($Sod2^{+/-}$ mice) exhibit increased oxidative damage and a higher incidence of cancer[109]. On the other hand, $Sod2^{+/-}$ mice and mice that overexpress SOD2 do not display altered life span when compared to littermate controls[109,110], suggesting that the antioxidant protection is important, but only one of the many enzymes important for protection against aging.

## Roles in Neuronal Cells and Brain

As mentioned earlier, in mammalian neuronal cells and in the CNS, the effect of the down-regulation of IGF-I signaling pathways is more complex than in simple model organisms. In fact, the signaling pathways and the related molecules described previously can play a dual role that can be, depending on the circumstances, beneficial or detrimental. This is true for the Ras and Akt pathways, and for ROS, whose production in neurons appears to be regulated also by Ras and Akt.

In neurons, Ras is involved in the cellular mechanisms underlying synaptic plasticity and memory formation[111], and neuronal polarity promotion, an essential event in neuronal differentiation[112]. In PC12 cells, superoxide can be generated by epidermal growth factor through a Ras- and MEK-dependent mechanism[113]. Ras is also required for activation of neuronal nitric oxide synthase in PC12 cells[114].

Akt is involved in neuronal differentiation and neurite outgrowth[115], can exert antioxidant effects on both the PNS and CNS[116,117], and can also protect against the disease-causing effects of peptides of β-amyloid proteins[118] and Parkinson-inducing toxins[119,120,121]. In addition, Akt plays antiapoptotic roles in neurons[122,123,124].

Among ROS active in the brain, nitric oxide and superoxide are essential in molecular processes underlying signal transduction, neuroprotection, synaptic plasticity, synaptogenesis, and memory formation (for reviews, see Calabrese[125] and Kishida and Klann[126], respectively). In particular, superoxide is necessary for long-term potentiation (LTP) induction, as proven by the fact that its application on hippocampal slices is sufficient to promote LTP in the CA1 area[127]. Moreover, mice lacking the NADPH oxidase proteins $gp91^{phox}$ and $p47^{phox}$, which play a crucial role in superoxide production, also lack LTP and exhibit mild impairment in hippocampus-dependent memory tasks, such as the Morris water maze and context-dependent fear-conditioning test[128]. Also, peroxynitrite, the product of the reaction between nitric oxide and superoxide, can provide a transient cytoprotection at low concentrations. In fact, pretreatment of primary neurons *in vitro* with peroxynitrite exerts transient neuroprotection against oxidative stress through activation of the PI3K/Akt pathway[129].

On the other hand, oncogenic mutations that cause the activation of the Ras or Akt pathways have been detected frequently in human cancers[121,130]. Mutations leading to a gain of function in the Ras signaling pathway can cause genetic disorders, such as neuro-cardio-facial-cutaneous (NCFC) and Hamartoma syndromes, characterized by mental retardation and behavioral disturbances[131]. Mice lacking sprouty-related proteins with an EVH-1 domain ($Spred1^{-/-}$ and $Spred1^{+/-}$ mice) and *Neurofibromatosis*-1 heterozygous mice ($Nf1^{+/-}$ mice), two models used in the study of NCFC syndromes, show learning and memory impairment, and defects in LTP and Ras pathway hyperactivation[132,133]. Notably, decreasing Ras activity in $Nf1^{+/-}$ mice reverses LTP deficits and cognitive problems[134].

Although short-term exposure to physiological levels of ROS in neurons is essential for neuronal function, prolonged exposure to high concentrations of radical species is harmful and can cause DNA damage, and possibly age-dependent mutations and cancer[135]. Notably, oxidative stress plays a major role in many neurodegenerative diseases[136,137,138], and age-dependent learning and memory impairments[139].

Reducing oxidative damage specifically in the brain can prevent age-dependent cognitive impairment. For example, SOD/catalase mimetics can reduce age-dependent protein oxidation and lipid peroxidation in mouse brains, and improve associative fear-conditioning task performances of aged mice[140]. Similarly, feeding aged rats with acetyl-*l*-carnitine (ALCAR) and lipoic acid (LA), two mitochondrial metabolites, improves performance in the Morris water maze by lowering oxidative damage in the hippocampus and improving mitochondrial functions[141]. Moreover, our studies suggested that nitric oxide and superoxide, through formation of their by-product peroxynitrite, are mediators of Aβ-activated microglia neurotoxicity in AD[142]. According to our findings, the lack of NADPH (nicotinamide adenine dinucleotide phosphate) oxidase protein $gp91^{phox}$ rescues the amyloid pathology mouse Tg2576 from neurovascular and cognitive dysfunctions[143].

GH and IGF-I in mammals are critical in brain maintenance and are involved in major aspects of the nervous system, such as neuronal development and plasticity[144,145]. However, in agreement with the proaging effect of the GH/IGF-I axis, mutations decreasing GH/IGF-I signaling can delay age-dependent memory impairment: for example, old Ames and Laron mice exhibited postponed cognitive impairment measured by inhibitory avoidance tests[146,147,148]. Among the explanations proposed to justify these findings are the local production of IGF-I at the hippocampus and the consequent activation of the antiapoptotic Akt signaling pathway that can contribute to neurogenesis[149,150]. Another explanation may be the improved insulin sensitivity exhibited by different long-lived mice that carry mutations in the GH/IGF-I, which can prevent type 2 diabetes and its associated cognitive impairment (for a review on diabetes mellitus and cognition, see Gispen and Biessels[151]).

Many studies support a beneficial role for CR in altering cognitive capabilities in mammals. Among the reasons proposed to be the cause of this effect are the reduction of oxidative stress in neurons, and the promotion of synaptic plasticity and induction of neurotrophic/neuroprotective factors as stress proteins[152,153]. Examples of positive CR effects collected from recent publications include stabilization of the age-dependent decrease of crucial synaptic proteins (such as GluR1 and GluR2 subunits of AMPA [alpha-amino-3-hydroxy-5-methyl-4-isoxazolepropionic acid] receptors) in the mouse CA3 and reduction of cognitive impairment[154], delay of age-dependent spatial memory impairment in old rats[155], and mitigation of the learning and memory deficits of a strain of senescence-accelerated prone mice[156].

A major neuroendocrinological effect caused by CR is induction of neuropeptide Y (NPY) in the arcuate nucleus (ARC). NPY has been associated with a number of processes, including anxiety, learning, and memory. Notably, recent studies have concluded that there is mechanistic separation between the CR and behavioral factors induced by NPY[157].

Also, recent findings suggest a possible correlation between CR and neurodegenerative diseases. For example, CR attenuates Aβ deposition in AD mice that carry mutations for APP (amyloid precursor protein) and APP + PS-1 (presenelin 1)[158], and ameliorates neurodegenerative phenotypes detected with object recognition and contextual fear-conditioning tests in cDKO (conditional double knockout) AD mice[159]. Interestingly, microarray analyses performed on the hippocampus of these AD mice have shown that CR could increase the expression of neurogenesis-related genes and decrease the expression of inflammation related genes in this brain region[159].

Recent studies performed in our laboratory have shown that short incubation in low-glucose or low-serum media protect primary rat glial cells, but not cancer cells, against $H_2O_2$ or the chemotherapy drug and oxidative stressor cyclophosphamide[160]. The study, based on our previous findings on enhanced resistance observed in long-lived yeast cells lacking *RAS2* and *SCH9,* suggests that this differential stress-resistance strategy can be used to protect the organism and likely also brain cells, but not cancer cells, against chemotherapy.

As discussed above, SIR2 proteins (or Sirtuins) may be involved in the CR process. Mammals possess seven sirtuins (SIRT 1–7)[36]. SIRT1 is the mammalian ortholog of yeast Sir2, and its orthologs in lower eukaryotes regulate crucial physiological processes, such as glucose metabolism, DNA repair, and apoptosis. Notably, SIRT1 is expressed at high levels in the brain, suggesting a role in the CNS[161,162]. In agreement with the findings carried out in lower eukaryotic species, sirtuins can play both protective and proaging roles in mammalian cells. Our recent investigations have linked SIRT1 activity to the activation of the IGF-I-Ras/ERK pathway and stress resistance in neurons[163]. Our studies suggest that inhibition of SIRT1 in rat neurons decreases the insulin/IGF-I–dependent activation of ERK1/2 by enhancing IRS-2 acetylation, and reducing IRS-2 phosphorylation and Ras activation. Either inhibition of SIRT1 or Ras is associated with increased stress resistance, and the brains of aged SIRT1-deficient mice display reduced oxidative damage. Interestingly, SIRT1 knockout mice are short-lived compared to wild-type controls under both normal and CR conditions, suggesting that antioxidant protection in neurons and in mouse brain is not translated into a longer life span, possibly because of developmental defects caused by the absence of SIRT1[163,164].

Recent studies have shown that, in both embryonic and adult neural precursor cells (NPCs), SIRT1 translocates from the cytoplasm to the nucleus in response to differentiation stimulus and that inhibition of SIRT1 activity significantly decreases neuronal differentiation[165]. These findings support a crucial role of SIRT1 in the modulation of neuronal differentiation.

Several reports have shown that SIRT1 activation can attenuate neuronal degeneration in animal models of neurodegenerative diseases (for reviews, see Tang and Chua[166] and Alcain and Villalba[167]). For example, different studies propose a possible neuroprotective role for SIRT1 in inhibiting axonal degeneration, an event common to neurodegenerative diseases such as PD and AD[168,169]. Other examples of positive beneficial effects related to SIRT1 activation in AD include the findings that SIRT1 overexpression, by repressing NF-kB signaling in microglia, protects against Aβ-mediated neurotoxicity in AD[170] and that in human brains affected by AD, SIRT1 reduction parallels the tau accumulation[171]. In addition, Pasinetti and coworkers showed that SIRT1 activation mediates FoxO3a inactivation, leading to preservation of spatial memory in the Tg2576 AD mouse[172].

However, recent papers have suggested that, at least under some conditions, SIRT1 inactivation, rather then activation, can provide cellular protection (for reviews see Tang[173] and Alcain and Villalba[174]). For example, the SIRT1 inhibitor nicotinamide can promote neuronal survival during acute anoxic injury[175]. In addition, when mouse embryonic stem cells are exposed to ROS, SIRT1 mediates apoptosis through p53 deacetylation and its translocation to mitochondria[176]. Recently, Hara and colleagues have shown that mice that overexpress SIRT1 (SIRT1 Tg) did not exhibit protection against neuronal damage induced by ischemia or the Parkinson-inducing neurotoxin MTPT[177]. However, SIRT1 Tg mice displayed memory deficits.

The protein kinase TOR, which, as mentioned above, is involved in the life span–extending effect of CR, is required for memory consolidation. In fact, the formation of long-term memory of one-trial inhibitory avoidance in rats is associated with a rapid increase in phosphorylation of mTOR and its substrate S6K[178]. Furthermore, novel results have shown that brain-derived neurotrophic factor (BDNF) is an upstream factor controlling mTOR signaling during memory consolidation in the hippocampus and that the increase of synaptic AMPA receptor GluR1, necessary for memory formation, is regulated by mTOR[179]. On the other hand, TOR inhibition has been shown to have beneficial effects on aggresome-related disease models, such as HD[180,181]. In addition, TOR signaling is involved in TSC syndrome, a disorder classified under Hamartoma syndromes[131]. The cognitive profile of TSC syndrome is variable and characterized by deficits in attention and memory and, in some cases, mental retardation. Studies performed on mice models developed for TSC reveal a direct role of TSC-1 and TSC-2, two proteins linked to the TOR signaling pathway (see Fig. 2), in synaptic plasticity[182].

## CONCLUSIONS

Over the past decade, numerous studies, a small part of which have been reviewed in this manuscript, have investigated the correlation between insulin/IGF signaling, and linked signaling pathways, longevity, oxidative stress, and neuronal/cognitive functions. The data discussed above illustrate the similarities among different organisms, and how the fundamental processes regulating stress resistance, cellular damage, and aging are substantially conserved through evolution, possibly extending to humans. In the light of these findings, the model organisms appear to be extraordinary models that, although very different from the human brain, can be exploited in order to study aging and to identify genes and pathways for protection against age-related neurodegeneration[183].

Insulin/IGF-I, TOR, S6 kinase, AC, PKA, and other genes converge and play partially overlapping roles in regulating aging and stress resistance in higher eukaryotes. The extremely complex signaling network of the mammalian brain appears to display many of the features observed in the model organisms, but neurobiology research in this area underlines that interventions to delay aging and prevent or treat neurodegenerative diseases will not be straightforward. The fact that signaling modulators from different pathways play different and sometimes opposing roles, depending on cellular and tissue condition, makes this topic challenging, but also extremely stimulating.

Although some aspects have already been investigated, the major part of the functional features and health status of long-lived mutant organisms, especially those linked to the nervous system and cognitive domain, remains largely unknown. Therefore, in the future, there is an urgent need for investigations aimed at studying crucial neuronal processes, such as neurogenesis, and to define the relationship between neuronal and glial aging, neurodegeneration, cognitive function, and longevity.

In the long run, the discoveries made in yeast, worms, flies, and mice will help in the identification and development of novel therapeutic strategies to treat natural age-related neurodegeneration and functional decline, as well as neurodegenerative diseases.

## References

1. Harman, D. (1956) Aging: a theory based on free radical and radiation chemistry. *J. Gerontol.* **11,** 298–300.
2. Martin, G.M., Austad, S.N., and Johnson, T.E. (1996) Genetic analysis of ageing: role of oxidative damage and environmental stresses. *Nat. Genet.* **13,** 25–34.
3. Poon, H.F., Calabrese, V., Scapagnini, G., and Butterfield, D.A. (2004) Free radicals and brain aging. *Clin. Geriatr. Med.* **20,** 329–359.
4. Liu, J. and Mori, A. (1999) Stress, aging, and brain oxidative damage. *Neurochem. Res.* **24,** 1479–1497.
5. Ames, B.N., Shigenaga, M.K., and Hagen, T.M. (1995) Mitochondrial decay in aging. *Biochim. Biophys. Acta* **1271,** 165–170.
6. Floyd, R.A. and Hensley, K. (2002) Oxidative stress in brain aging. Implications for therapeutics of neurodegenerative diseases. *Neurobiol. Aging* **23,** 795–807.
7. Forster, M.J., Dubey, A., Dawson, K.M., Stutts, W.A., Lal, H., and Sohal, R.S. (1996) Age-related losses of cognitive function and motor skills in mice are associated with oxidative protein damage in the brain. *Proc. Natl. Acad. Sci. U. S. A.* **93,** 4765–4769.
8. Mecocci, P., MacGarvey, U., Kaufman, A.E., Koontz, D., Shoffner, J.M., Wallace, D.C., and Beal, M.F. (1993) Oxidative damage to mitochondrial DNA shows marked age-dependent increases in human brain. *Ann. Neurol.* **34,** 609–616.
9. Stadtman, E.R. (1992) Protein oxidation and aging. *Science* **257,** 1220–1224.
10. Montine, T.J., Neely, M.D., Quinn, J.F., Beal, M.F., Markesbery, W.R., Roberts, L.J., and Morrow, J.D. (2002) Lipid peroxidation in aging brain and Alzheimer's disease. *Free Radic. Biol. Med.* **33,** 620–626.
11. Pratico, D. and Delanty, N. (2000) Oxidative injury in diseases of the central nervous system: focus on Alzheimer's disease. *Am. J. Med.* **109,** 577–585.
12. Thomas, B. and Beal, M.F. (2007) Parkinson's disease. *Hum. Mol. Genet.* **16(Spec No. 2),** R183–194.
13. Stack, E.C. and Ferrante, R.J. (2007) Huntington's disease: progress and potential in the field. *Expert Opin. Investig. Drugs* **16,** 1933–1953.
14. Leutner, S., Czech, C., Schindowski, K., Touchet, N., Eckert, A., and Muller, W.E. (2000) Reduced antioxidant enzyme activity in brains of mice transgenic for human presenilin-1 with single or multiple mutations. *Neurosci. Lett.* **292,** 87–90.
15. Schuessel, K., Frey, C., Jourdan, C., Keil, U., Weber, C.C., Muller-Spahn, F., Muller, W.E., and Eckert, A. (2006) Aging sensitizes toward ROS formation and lipid peroxidation in PS1M146L transgenic mice. *Free Radic. Biol. Med.* **40,** 850–862.
16. Longo, V.D. and Finch, C.E. (2003) Evolutionary medicine: from dwarf model systems to healthy centenarians? *Science* **299,** 1342–1346.
17. Harper, J.M., Salmon, A.B., Chang, Y., Bonkowski, M., Bartke, A., and Miller, R.A. (2006) Stress resistance and aging: influence of genes and nutrition. *Mech. Ageing Dev.* **127,** 687–694.
18. Parrella, E. and Longo, V.D. (2008) The chronological life span of Saccharomyces cerevisiae to study mitochondrial dysfunction and disease. *Methods* **46,** 256–262.
19. Fabrizio, P., Liou, L.L., Moy, V.N., Diaspro, A., Valentine, J.S., Gralla, E.B., and Longo, V.D. (2003) SOD2 functions downstream of Sch9 to extend longevity in yeast. *Genetics* **163,** 35–46.
20. Longo, V.D., Gralla, E.B., and Valentine, J.S. (1996) Superoxide dismutase activity is essential for stationary phase survival in Saccharomyces cerevisiae. Mitochondrial production of toxic oxygen species in vivo. *J. Biol. Chem.* **271,** 12275–12280.
21. Longo, V.D., Liou, L.L., Valentine, J.S., and Gralla, E.B. (1999) Mitochondrial superoxide decreases yeast survival in stationary phase. *Arch. Biochem. Biophys.* **365,** 131–142.
22. Kaeberlein, M., Powers, R.W., 3rd, Steffen, K.K., Westman, E.A., Hu, D., Dang, N., Kerr, E.O., Kirkland, K.T., Fields, S., and Kennedy, B.K. (2005) Regulation of yeast replicative life span by TOR and Sch9 in response to nutrients. *Science* **310,** 1193–1196.
23. Urban, J., Soulard, A., Huber, A., Lippman, S., Mukhopadhyay, D., Deloche, O., Wanke, V., Anrather, D., Ammerer, G., Riezman, H., Broach, J.R., De Virgilio, C., Hall, M.N., and Loewith, R. (2007) Sch9 is a major target of TORC1 in Saccharomyces cerevisiae. *Mol. Cell* **26,** 663–674.
24. Toda, T., Cameron, S., Sass, P., and Wigler, M. (1988) SCH9, a gene of Saccharomyces cerevisiae that encodes a protein distinct from, but functionally and structurally related to, cAMP-dependent protein kinase catalytic subunits. *Genes Dev.* **2,** 517–527.
25. Fabrizio, P., Pozza, F., Pletcher, S.D., Gendron, C.M., and Longo, V.D. (2001) Regulation of longevity and stress resistance by Sch9 in yeast. *Science* **292,** 288–290.
26. Kaeberlein, M., Hu, D., Kerr, E.O., Tsuchiya, M., Westman, E.A., Dang, N., Fields, S., and Kennedy, B.K. (2005) Increased life span due to calorie restriction in respiratory-deficient yeast. *PLoS Genet.* **1,** e69.
27. Kaeberlein, M. and Kennedy, B.K. (2005) Large-scale identification in yeast of conserved ageing genes. *Mech. Ageing Dev.* **126,** 17–21.
28. Bonawitz, N.D., Chatenay-Lapointe, M., Pan, Y., and Shadel, G.S. (2007) Reduced TOR signaling extends chronological life span via increased respiration and upregulation of mitochondrial gene expression. *Cell Metab.* **5,** 265–277.
29. Powers, R.W., 3rd, Kaeberlein, M., Caldwell, S.D., Kennedy, B.K., and Fields, S. (2006) Extension of chronological life span in yeast by decreased TOR pathway signaling. *Genes Dev.* **20,** 174–184.
30. Longo, V.D. (2004) Ras: the other pro-aging pathway. *Sci. Aging Knowledge Environ.* **2004,** pe36.
31. Longo, V.D. (1999) Mutations in signal transduction proteins increase stress resistance and longevity in yeast, nematodes, fruit flies, and mammalian neuronal cells. *Neurobiol. Aging* **20,** 479–486.
32. Longo, V.D. and Fabrizio, P. (2002) Regulation of longevity and stress resistance: a molecular strategy conserved from yeast to humans? *Cell. Mol. Life Sci.* **59,** 903–908.
33. Wei, M., Fabrizio, P., Madia, F., Hu, J., Ge, H., Li, L.M., and Longo, V.D. (2009) Tor1/Sch9-regulated carbon source substitution is as effective as calorie restriction in life span extension. *PLoS Genet.* **5,** e1000467.
34. Longo, V.D. (2009) Linking sirtuins, IGF-I signaling, and starvation. *Exp. Gerontol.* **44,** 70–74.
35. Longo, V.D. and Kennedy, B.K. (2006) Sirtuins in aging and age-related disease. *Cell* **126,** 257–268.
36. Blander, G. and Guarente, L. (2004) The Sir2 family of protein deacetylases. *Annu. Rev. Biochem.* **73,** 417–435.
37. Lin, S.J., Defossez, P.A., and Guarente, L. (2000) Requirement of NAD and SIR2 for life-span extension by calorie restriction in Saccharomyces cerevisiae. *Science* **289,** 2126–2128.
38. Guarente, L. and Picard, F. (2005) Calorie restriction--the SIR2 connection. *Cell* **120,** 473–482.
39. Kaeberlein, M., Kirkland, K.T., Fields, S., and Kennedy, B.K. (2004) Sir2-independent life span extension by calorie restriction in yeast. *PLoS Biol.* **2,** E296.
40. Fabrizio, P., Gattazzo, C., Battistella, L., Wei, M., Cheng, C., McGrew, K., and Longo, V.D. (2005) Sir2 blocks extreme life-span extension. *Cell* **123,** 655–667.
41. Kenyon, C., Chang, J., Gensch, E., Rudner, A., and Tabtiang, R. (1993) A C. elegans mutant that lives twice as long as wild type. *Nature* **366,** 461–464.
42. Kimura, K.D., Tissenbaum, H.A., Liu, Y., and Ruvkun, G. (1997) daf-2, an insulin receptor-like gene that regulates longevity and diapause in Caenorhabditis elegans. *Science* **277,** 942–946.
43. Lin, K., Dorman, J.B., Rodan, A., and Kenyon, C. (1997) daf-16: an HNF-3/forkhead family member that can function to double the life-span of Caenorhabditis elegans. *Science* **278,** 1319–1322.
44. Ogg, S., Paradis, S., Gottlieb, S., Patterson, G.I., Lee, L., Tissenbaum, H.A., and Ruvkun, G. (1997) The Fork head transcription factor DAF-16 transduces insulin-like metabolic and longevity signals in C. elegans. *Nature* **389,** 994–999.
45. Friedman, D.B. and Johnson, T.E. (1988) A mutation in the age-1 gene in Caenorhabditis elegans lengthens life and reduces hermaphrodite fertility. *Genetics* **118,** 75–86.
46. Klass, M.R. (1983) A method for the isolation of longevity mutants in the nematode Caenorhabditis elegans and initial results. *Mech. Ageing Dev.* **22,** 279–286.
47. Morris, J.Z., Tissenbaum, H.A., and Ruvkun, G. (1996) A phosphatidylinositol-3-OH kinase family member regulating longevity and diapause in Caenorhabditis elegans. *Nature* **382,** 536–539.
48. Guarente, L. and Kenyon, C. (2000) Genetic pathways that regulate ageing in model organisms. *Nature* **408,** 255–262.
49. Paradis, S., Ailion, M., Toker, A., Thomas, J.H., and Ruvkun, G. (1999) A PDK1 homolog is necessary and sufficient to transduce AGE-1 PI3 kinase signals that regulate diapause in Caenorhabditis elegans. *Genes Dev.* **13,** 1438–1452.
50. Pan, K.Z., Palter, J.E., Rogers, A.N., Olsen, A., Chen, D., Lithgow, G.J., and Kapahi, P. (2007) Inhibition of mRNA translation extends lifespan in Caenorhabditis elegans. *Aging Cell* **6,** 111–119.
51. Honda, Y. and Honda, S. (1999) The daf-2 gene network for longevity regulates oxidative stress resistance and Mn-superoxide dismutase gene expression in Caenorhabditis elegans. *FASEB J.* **13,** 1385–1393.
52. Murphy, C.T., McCarroll, S.A., Bargmann, C.I., Fraser, A., Kamath, R.S., Ahringer, J., Li, H., and Kenyon, C. (2003)

Genes that act downstream of DAF-16 to influence the lifespan of Caenorhabditis elegans. *Nature* **424,** 277–283.
53. Cherkasova, V., Ayyadevara, S., Egilmez, N., and Shmookler Reis, R. (2000) Diverse Caenorhabditis elegans genes that are upregulated in dauer larvae also show elevated transcript levels in long-lived, aged, or starved adults. *J. Mol. Biol.* **300,** 433–448.
54. McElwee, J.J., Schuster, E., Blanc, E., Thomas, J.H., and Gems, D. (2004) Shared transcriptional signature in Caenorhabditis elegans Dauer larvae and long-lived daf-2 mutants implicates detoxification system in longevity assurance. *J. Biol. Chem.* **279,** 44533–44543.
55. Sampayo, J.N., Olsen, A., and Lithgow, G.J. (2003) Oxidative stress in Caenorhabditis elegans: protective effects of superoxide dismutase/catalase mimetics. *Aging Cell* **2,** 319–326.
56. Jia, K., Chen, D., and Riddle, D.L. (2004) The TOR pathway interacts with the insulin signaling pathway to regulate C. elegans larval development, metabolism and life span. *Development* **131,** 3897–3906.
57. Vellai, T., Takacs-Vellai, K., Zhang, Y., Kovacs, A.L., Orosz, L., and Muller, F. (2003) Genetics: influence of TOR kinase on lifespan in C. elegans. *Nature* **426,** 620.
58. Houthoofd, K. and Vanfleteren, J.R. (2006) The longevity effect of dietary restriction in Caenorhabditis elegans. *Exp. Gerontol.* **41,** 1026–1031.
59. Schulz, T.J., Zarse, K., Voigt, A., Urban, N., Birringer, M., and Ristow, M. (2007) Glucose restriction extends Caenorhabditis elegans life span by inducing mitochondrial respiration and increasing oxidative stress. *Cell Metab.* **6,** 280–293.
60. Garsin, D.A., Villanueva, J.M., Begun, J., Kim, D.H., Sifri, C.D., Calderwood, S.B., Ruvkun, G., and Ausubel, F.M. (2003) Long-lived C. elegans daf-2 mutants are resistant to bacterial pathogens. *Science* **300,** 1921.
61. Huang, C., Xiong, C., and Kornfeld, K. (2004) Measurements of age-related changes of physiological processes that predict lifespan of Caenorhabditis elegans. *Proc. Natl. Acad. Sci. U. S. A.* **101,** 8084–8089.
62. Libina, N., Berman, J.R., and Kenyon, C. (2003) Tissue-specific activities of C. elegans DAF-16 in the regulation of lifespan. *Cell* **115,** 489–502.
63. Wolkow, C.A., Kimura, K.D., Lee, M.S., and Ruvkun, G. (2000) Regulation of C. elegans life-span by insulinlike signaling in the nervous system. *Science* **290,** 147–150.
64. Herndon, L.A., Schmeissner, P.J., Dudaronek, J.M., Brown, P.A., Listner, K.M., Sakano, Y., Paupard, M.C., Hall, D.H., and Driscoll, M. (2002) Stochastic and genetic factors influence tissue-specific decline in ageing C. elegans. *Nature* **419,** 808–814.
65. Murakami, S. (2007) Caenorhabditis elegans as a model system to study aging of learning and memory. *Mol. Neurobiol.* **35,** 85–94.
66. Apfeld, J. and Kenyon, C. (1999) Regulation of lifespan by sensory perception in Caenorhabditis elegans. *Nature* **402,** 804–809.
67. Fujii, M., Matsumoto, Y., Tanaka, N., Miki, K., Suzuki, T., Ishii, N., and Ayusawa, D. (2004) Mutations in chemosensory cilia cause resistance to paraquat in nematode Caenorhabditis elegans. *J. Biol. Chem.* **279,** 20277–20282.
68. Clancy, D.J., Gems, D., Harshman, L.G., Oldham, S., Stocker, H., Hafen, E., Leevers, S.J., and Partridge, L. (2001) Extension of life-span by loss of CHICO, a Drosophila insulin receptor substrate protein. *Science* **292,** 104–106.
69. Tatar, M., Kopelman, A., Epstein, D., Tu, M.P., Yin, C.M., and Garofalo, R.S. (2001) A mutant Drosophila insulin receptor homolog that extends life-span and impairs neuroendocrine function. *Science* **292,** 107–110.
70. Wessells, R.J., Fitzgerald, E., Cypser, J.R., Tatar, M., and Bodmer, R. (2004) Insulin regulation of heart function in aging fruit flies. *Nat. Genet.* **36,** 1275–1281.
71. Kapahi, P., Zid, B.M., Harper, T., Koslover, D., Sapin, V., and Benzer, S. (2004) Regulation of lifespan in Drosophila by modulation of genes in the TOR signaling pathway. *Curr. Biol.* **14,** 885–890.
72. Rogina, B. and Helfand, S.L. (2004) Sir2 mediates longevity in the fly through a pathway related to calorie restriction. *Proc. Natl. Acad. Sci. U. S. A.* **101,** 15998–16003.
73. Tatar, M. (2007) Diet restriction in Drosophila melanogaster. Design and analysis. *Interdiscip. Top. Gerontol.* **35,** 115–136.
74. Piper, M.D. and Partridge, L. (2007) Dietary restriction in Drosophila: delayed aging or experimental artefact? *PLoS Genet.* **3,** e57.
75. Agarwal, S. and Sohal, R.S. (1994) DNA oxidative damage and life expectancy in houseflies. *Proc. Natl. Acad. Sci. U. S. A.* **91,** 12332–12335.
76. Agarwal, S. and Sohal, R.S. (1994) Aging and protein oxidative damage. *Mech. Ageing Dev.* **75,** 11–19.
77. Sun, J., Folk, D., Bradley, T.J., and Tower, J. (2002) Induced overexpression of mitochondrial Mn-superoxide dismutase extends the life span of adult Drosophila melanogaster. *Genetics* **161,** 661–672.
78. Parkes, T.L., Elia, A.J., Dickinson, D., Hilliker, A.J., Phillips, J.P., and Boulianne, G.L. (1998) Extension of Drosophila lifespan by overexpression of human SOD1 in motorneurons. *Nat. Genet.* **19,** 171–174.
79. Sun, J. and Tower, J. (1999) FLP recombinase-mediated induction of Cu/Zn-superoxide dismutase transgene expression can extend the life span of adult Drosophila melanogaster flies. *Mol. Cell. Biol.* **19,** 216–228.
80. Magwere, T., West, M., Riyahi, K., Murphy, M.P., Smith, R.A., and Partridge, L. (2006) The effects of exogenous antioxidants on lifespan and oxidative stress resistance in Drosophila melanogaster. *Mech. Ageing Dev.* **127,** 356–370.
81. Yan, L.J., Levine, R.L., and Sohal, R.S. (1997) Oxidative damage during aging targets mitochondrial aconitase. *Proc. Natl. Acad. Sci. U. S. A.* **94,** 11168–11172.

82. Martin, I., Jones, M.A., and Grotewiel, M. (2009) Manipulation of Sod1 expression ubiquitously, but not in the nervous system or muscle, impacts age-related parameters in Drosophila. *FEBS Lett.* **583(13),** 2308–2314.
83. Martin, I., Jones, M.A., Rhodenizer, D., Zheng, J., Warrick, J.M., Seroude, L., and Grotewiel, M. (2009) Sod2 knock-down in the musculature has whole organism consequences in Drosophila. *Free Radic. Biol. Med.* **47(6),** 803–813.
84. Piazza, N., Hayes, M., Martin, I., Duttaroy, A., Grotewiel, M., and Wessells, R. (2009) Multiple measures of functionality exhibit progressive decline in a parallel, stochastic fashion in Drosophila Sod2 null mutants. *Biogerontology* **10(5),** 637–648.
85. Orr, W.C. and Sohal, R.S. (1993) Effects of Cu-Zn superoxide dismutase overexpression of life span and resistance to oxidative stress in transgenic Drosophila melanogaster. *Arch. Biochem. Biophys.* **301,** 34–40.
86. Seto, N.O., Hayashi, S., and Tener, G.M. (1990) Overexpression of Cu-Zn superoxide dismutase in Drosophila does not affect life-span. *Proc. Natl. Acad. Sci. U. S. A.* **87,** 4270–4274.
87. Orr, W.C. and Sohal, R.S. (2003) Does overexpression of Cu,Zn-SOD extend life span in Drosophila melanogaster? *Exp. Gerontol.* **38,** 227–230.
88. Horiuchi, J. and Saitoe, M. (2005) Can flies shed light on our own age-related memory impairment? *Ageing Res. Rev.* **4,** 83–101.
89. Neckameyer, W.S., Woodrome, S., Holt, B., and Mayer, A. (2000) Dopamine and senescence in Drosophila melanogaster. *Neurobiol. Aging* **21,** 145–152.
90. Martin, I. and Grotewiel, M.S. (2006) Distinct genetic influences on locomotor senescence in Drosophila revealed by a series of metrical analyses. *Exp. Gerontol.* **41,** 877–881.
91. Jones, M.A., Gargano, J.W., Rhodenizer, D., Martin, I., Bhandari, P., and Grotewiel, M. (2009) A forward genetic screen in Drosophila implicates insulin signaling in age-related locomotor impairment. *Exp. Gerontol.* **44(8)**, 532–40.
92. Sanchez, D., Lopez-Arias, B., Torroja, L., Canal, I., Wang, X., Bastiani, M.J., and Ganfornina, M.D. (2006) Loss of glial lazarillo, a homolog of apolipoprotein D, reduces lifespan and stress resistance in Drosophila. *Curr. Biol.* **16,** 680–686.
93. Walker, D.W., Muffat, J., Rundel, C., and Benzer, S. (2006) Overexpression of a Drosophila homolog of apolipoprotein D leads to increased stress resistance and extended lifespan. *Curr. Biol.* **16,** 674–679.
94. Berryman, D.E., Christiansen, J.S., Johannsson, G., Thorner, M.O., and Kopchick, J.J. (2008) Role of the GH/IGF-1 axis in lifespan and healthspan: lessons from animal models. *Growth Horm. IGF Res.* **18,** 455–471.
95. Brown-Borg, H.M., Borg, K.E., Meliska, C.J., and Bartke, A. (1996) Dwarf mice and the ageing process. *Nature* **384**, 33.
96. Bartke, A., Wright, J.C., Mattison, J.A., Ingram, D.K., Miller, R.A., and Roth, G.S. (2001) Extending the lifespan of long-lived mice. *Nature* **414,** 412.
97. Flurkey, K., Papaconstantinou, J., Miller, R.A., and Harrison, D.E. (2001) Lifespan extension and delayed immune and collagen aging in mutant mice with defects in growth hormone production. *Proc. Natl. Acad. Sci. U. S. A.* **98,** 6736–6741.
98. Zhou, Y., Xu, B.C., Maheshwari, H.G., He, L., Reed, M., Lozykowski, M., Okada, S., Cataldo, L., Coschigamo, K., Wagner, T.E., Baumann, G., and Kopchick, J.J. (1997) A mammalian model for Laron syndrome produced by targeted disruption of the mouse growth hormone receptor/binding protein gene (the Laron mouse). *Proc. Natl. Acad. Sci. U. S. A.* **94,** 13215–13220.
99. Holzenberger, M., Dupont, J., Ducos, B., Leneuve, P., Geloen, A., Even, P.C., Cervera, P., and Le Bouc, Y. (2003) IGF-1 receptor regulates lifespan and resistance to oxidative stress in mice. *Nature* **421,** 182–187.
100. Richardson, A., Liu, F., Adamo, M.L., Van Remmen, H., and Nelson, J.F. (2004) The role of insulin and insulin-like growth factor-I in mammalian ageing. *Best Pract. Res. Clin. Endocrinol. Metab.* **18,** 393–406.
101. Selman, C., Lingard, S., Choudhury, A.I., Batterham, R.L., Claret, M., Clements, M., Ramadani, F., Okkenhaug, K., Schuster, E., Blanc, E., Piper, M.D., Al-Qassab, H., Speakman, J.R., Carmignac, D., Robinson, I.C., Thornton, J.M., Gems, D., Partridge, L., and Withers, D.J. (2008) Evidence for lifespan extension and delayed age-related biomarkers in insulin receptor substrate 1 null mice. *FASEB J.* **22,** 807–818.
102. Taguchi, A., Wartschow, L.M., and White, M.F. (2007) Brain IRS2 signaling coordinates life span and nutrient homeostasis. *Science* **317,** 369–372.
103. Coschigano, K.T., Holland, A.N., Riders, M.E., List, E.O., Flyvbjerg, A., and Kopchick, J.J. (2003) Deletion, but not antagonism, of the mouse growth hormone receptor results in severely decreased body weights, insulin, and insulin-like growth factor I levels and increased life span. *Endocrinology* **144,** 3799–3810.
104. Raught, B., Gingras, A.C., and Sonenberg, N. (2001) The target of rapamycin (TOR) proteins. *Proc. Natl. Acad. Sci. U. S. A.* **98,** 7037–7044.
105. Brown-Borg, H.M. and Rakoczy, S.G. (2000) Catalase expression in delayed and premature aging mouse models. *Exp. Gerontol.* **35,** 199–212.
106. Brown-Borg, H.M., Rakoczy, S.G., Romanick, M.A., and Kennedy, M.A. (2002) Effects of growth hormone and insulin-like growth factor-1 on hepatocyte antioxidative enzymes. *Exp. Biol. Med. (Maywood)* **227,** 94–104.
107. Li, Y., Huang, T.T., Carlson, E.J., Melov, S., Ursell, P.C., Olson, J.L., Noble, L.J., Yoshimura, M.P., Berger, C., Chan, P.H., Wallace, D.C., and Epstein, C.J. (1995) Dilated cardiomyopathy and neonatal lethality in mutant mice lacking manganese superoxide dismutase. *Nat. Genet.* **11,** 376–381.
108. Lebovitz, R.M., Zhang, H., Vogel, H., Cartwright, J., Jr., Dionne, L., Lu, N., Huang, S., and Matzuk, M.M. (1996) Neurodegeneration, myocardial injury, and perinatal death in mitochondrial superoxide dismutase-deficient mice.

*Proc. Natl. Acad. Sci. U. S. A.* **93,** 9782–9787.

109. Van Remmen, H., Ikeno, Y., Hamilton, M., Pahlavani, M., Wolf, N., Thorpe, S.R., Alderson, N.L., Baynes, J.W., Epstein, C.J., Huang, T.T., Nelson, J., Strong, R., and Richardson, A. (2003) Life-long reduction in MnSOD activity results in increased DNA damage and higher incidence of cancer but does not accelerate aging. *Physiol. Genomics* **16,** 29–37.
110. Jang, Y.C., Perez, V.I., Song, W., Lustgarten, M.S., Salmon, A.B., Mele, J., Qi, W., Liu, Y., Liang, H., Chaudhuri, A., Ikeno, Y., Epstein, C.J., Van Remmen, H., and Richardson, A. (2009) Overexpression of Mn superoxide dismutase does not increase life span in mice. *J. Gerontol. A Biol. Sci. Med. Sci.* **64(11),** 1114–1125.
111. Selcher, J.C., Atkins, C.M., Trzaskos, J.M., Paylor, R., and Sweatt, J.D. (1999) A necessity for MAP kinase activation in mammalian spatial learning. *Learn. Mem.* **6,** 478–490.
112. Yoshimura, T., Arimura, N., Kawano, Y., Kawabata, S., Wang, S., and Kaibuchi, K. (2006) Ras regulates neuronal polarity via the PI3-kinase/Akt/GSK-3beta/CRMP-2 pathway. *Biochem. Biophys. Res. Commun.* **340,** 62–68.
113. Mills, E.M., Takeda, K., Yu, Z.X., Ferrans, V., Katagiri, Y., Jiang, H., Lavigne, M.C., Leto, T.L., and Guroff, G. (1998) Nerve growth factor treatment prevents the increase in superoxide produced by epidermal growth factor in PC12 cells. *J. Biol. Chem*. **273,** 22165–22168.
114. Schonhoff, C.M., Bulseco, D.A., Brancho, D.M., Parada, L.F., and Ross, A.H. (2001) The Ras-ERK pathway is required for the induction of neuronal nitric oxide synthase in differentiating PC12 cells. *J. Neurochem.* **78,** 631–639.
115. Read, D.E. and Gorman, A.M. (2009) Involvement of Akt in neurite outgrowth. *Cell. Mol. Life Sci.* **66(18),** 2975–2984.
116. Brunet, A., Datta, S.R., and Greenberg, M.E. (2001) Transcription-dependent and -independent control of neuronal survival by the PI3K-Akt signaling pathway. *Curr. Opin. Neurobiol.* **11,** 297–305.
117. Franke, T.F., Hornik, C.P., Segev, L., Shostak, G.A., and Sugimoto, C. (2003) PI3K/Akt and apoptosis: size matters. *Oncogene* **22,** 8983–8998.
118. Martin, D., Salinas, M., Lopez-Valdaliso, R., Serrano, E., Recuero, M., and Cuadrado, A. (2001) Effect of the Alzheimer amyloid fragment Abeta(25-35) on Akt/PKB kinase and survival of PC12 cells. *J. Neurochem.* **78,** 1000–1008.
119. Salinas, M., Diaz, R., Abraham, N.G., Ruiz de Galarreta, C.M., and Cuadrado, A. (2003) Nerve growth factor protects against 6-hydroxydopamine-induced oxidative stress by increasing expression of heme oxygenase-1 in a phosphatidylinositol 3-kinase-dependent manner. *J. Biol. Chem.* **278,** 13898–13904.
120. Salinas, M., Martin, D., Alvarez, A., and Cuadrado, A. (2001) Akt1/PKBalpha protects PC12 cells against the parkinsonism-inducing neurotoxin 1-methyl-4-phenylpyridinium and reduces the levels of oxygen-free radicals. *Mol. Cell. Neurosci.* **17,** 67–77.
121. Toker, A. and Yoeli-Lerner, M. (2006) Akt signaling and cancer: surviving but not moving on. *Cancer Res.* **66,** 3963–3966.
122. Dudek, H., Datta, S.R., Franke, T.F., Birnbaum, M.J., Yao, R., Cooper, G.M., Segal, R.A., Kaplan, D.R., and Greenberg, M.E. (1997) Regulation of neuronal survival by the serine-threonine protein kinase Akt. *Science* **275,** 661–665.
123. Martin, D., Salinas, M., Fujita, N., Tsuruo, T., and Cuadrado, A. (2002) Ceramide and reactive oxygen species generated by H2O2 induce caspase-3-independent degradation of Akt/protein kinase B. *J. Biol. Chem.* **277,** 42943–42952.
124. Erlich, S., Goldshmit, Y., Lupowitz, Z., and Pinkas-Kramarski, R. (2001) ErbB-4 activation inhibits apoptosis in PC12 cells. *Neuroscience* **107,** 353–362.
125. Calabrese, V., Mancuso, C., Calvani, M., Rizzarelli, E., Butterfield, D.A., and Stella, A.M. (2007) Nitric oxide in the central nervous system: neuroprotection versus neurotoxicity. *Nat. Rev. Neurosci*. **8,** 766–775.
126. Kishida, K.T. and Klann, E. (2007) Sources and targets of reactive oxygen species in synaptic plasticity and memory. *Antioxid. Redox Signal.* **9,** 233–244.
127. Huddleston, A.T., Tang, W., Takeshima, H., Hamilton, S.L., and Klann, E. (2008) Superoxide-induced potentiation in the hippocampus requires activation of ryanodine receptor type 3 and ERK. *J. Neurophysiol.* **99,** 1565–1571.
128. Kishida, K.T., Hoeffer, C.A., Hu, D., Pao, M., Holland, S.M., and Klann, E. (2006) Synaptic plasticity deficits and mild memory impairments in mouse models of chronic granulomatous disease. *Mol. Cell. Biol.* **26,** 5908–5920.
129. Delgado-Esteban, M., Martin-Zanca, D., Andres-Martin, L., Almeida, A., and Bolanos, J.P. (2007) Inhibition of PTEN by peroxynitrite activates the phosphoinositide-3-kinase/Akt neuroprotective signaling pathway. *J. Neurochem.* **102,** 194–205.
130. Rodriguez-Viciana, P., Tetsu, O., Oda, K., Okada, J., Rauen, K., and McCormick, F. (2005) Cancer targets in the Ras pathway. *Cold Spring Harb. Symp. Quant. Biol.* **70,** 461–467.
131. Krab, L.C., Goorden, S.M., and Elgersma, Y. (2008) Oncogenes on my mind: ERK and MTOR signaling in cognitive diseases. *Trends Genet.* **24,** 498–510.
132. Costa, R.M., Federov, N.B., Kogan, J.H., Murphy, G.G., Stern, J., Ohno, M., Kucherlapati, R., Jacks, T., and Silva, A.J. (2002) Mechanism for the learning deficits in a mouse model of neurofibromatosis type 1. *Nature* **415,** 526–530.
133. Denayer, E., Ahmed, T., Brems, H., Van Woerden, G., Borgesius, N.Z., Callaerts-Vegh, Z., Yoshimura, A., Hartmann, D., Elgersma, Y., D'Hooge, R., Legius, E., and Balschun, D. (2008) Spred1 is required for synaptic plasticity and hippocampus-dependent learning. *J. Neurosci.* **28,** 14443–14449.
134. Li, W., Cui, Y., Kushner, S.A., Brown, R.A., Jentsch, J.D., Frankland, P.W., Cannon, T.D., and Silva, A.J. (2005) The HMG-CoA reductase inhibitor lovastatin reverses the learning and attention deficits in a mouse model of neurofibromatosis type 1. *Curr. Biol.* **15,** 1961–1967.

135. Longo, V.D., Lieber, M.R., and Vijg, J. (2008) Turning anti-ageing genes against cancer. *Nat. Rev. Mol. Cell Biol.* **9,** 903–910.
136. Zhou, C., Huang, Y., and Przedborski, S. (2008) Oxidative stress in Parkinson's disease: a mechanism of pathogenic and therapeutic significance. *Ann. N. Y. Acad. Sci*. **1147,** 93–104.
137. Zawia, N.H., Lahiri, D.K., and Cardozo-Pelaez, F. (2009) Epigenetics, oxidative stress, and Alzheimer disease. *Free Radic. Biol. Med.* **46,** 1241–1249.
138. Lovell, M.A. and Markesbery, W.R. (2007) Oxidative DNA damage in mild cognitive impairment and late-stage Alzheimer's disease. *Nucleic Acids Res.* **35,** 7497–7504.
139. Asha Devi, S. (2009) Aging brain: prevention of oxidative stress by vitamin E and exercise. *TheScientificWorldJOURNAL* **9,** 366–372.
140. Liu, R., Liu, I.Y., Bi, X., Thompson, R.F., Doctrow, S.R., Malfroy, B., and Baudry, M. (2003) Reversal of age-related learning deficits and brain oxidative stress in mice with superoxide dismutase/catalase mimetics. *Proc. Natl. Acad. Sci. U. S. A.* **100,** 8526–8531.
141. Liu, J., Head, E., Gharib, A.M., Yuan, W., Ingersoll, R.T., Hagen, T.M., Cotman, C.W., and Ames, B.N. (2002) Memory loss in old rats is associated with brain mitochondrial decay and RNA/DNA oxidation: partial reversal by feeding acetyl-L-carnitine and/or R-alpha -lipoic acid. *Proc. Natl. Acad. Sci. U. S. A.* **99,** 2356–2361.
142. Xie, Z., Wei, M., Morgan, T.E., Fabrizio, P., Han, D., Finch, C.E., and Longo, V.D. (2002) Peroxynitrite mediates neurotoxicity of amyloid beta-peptide1-42- and lipopolysaccharide-activated microglia. *J. Neurosci.* **22**, 3484–3492.
143. Park, L., Zhou, P., Pitstick, R., Capone, C., Anrather, J., Norris, E.H., Younkin, L., Younkin, S., Carlson, G., McEwen, B.S., and Iadecola, C. (2008) Nox2-derived radicals contribute to neurovascular and behavioral dysfunction in mice overexpressing the amyloid precursor protein. *Proc. Natl. Acad. Sci. U. S. A.* **105,** 1347–1352.
144. van Dam, P.S. and Aleman, A. (2004) Insulin-like growth factor-I, cognition and brain aging. *Eur. J. Pharmacol.* **490,** 87–95.
145. Aberg, N.D., Brywe, K.G., and Isgaard, J. (2006) Aspects of growth hormone and insulin-like growth factor-I related to neuroprotection, regeneration, and functional plasticity in the adult brain. *TheScientificWorldJOURNAL* **6,** 53–80.
146. Kinney, B.A., Coschigano, K.T., Kopchick, J.J., Steger, R.W., and Bartke, A. (2001) Evidence that age-induced decline in memory retention is delayed in growth hormone resistant GH-R-KO (Laron) mice. *Physiol. Behav.* **72,** 653–660.
147. Kinney, B.A., Meliska, C.J., Steger, R.W., and Bartke, A. (2001) Evidence that Ames dwarf mice age differently from their normal siblings in behavioral and learning and memory parameters. *Horm. Behav.* **39,** 277–284.
148. Kinney-Forshee, B.A., Kinney, N.E., Steger, R.W., and Bartke, A. (2004) Could a deficiency in growth hormone signaling be beneficial to the aging brain? *Physiol. Behav.* **80,** 589–594.
149. Sun, L.Y., Al-Regaiey, K., Masternak, M.M., Wang, J., and Bartke, A. (2005) Local expression of GH and IGF-1 in the hippocampus of GH-deficient long-lived mice. *Neurobiol. Aging* **26,** 929–937.
150. Sun, L.Y. and Bartke, A. (2007) Adult neurogenesis in the hippocampus of long-lived mice during aging. *J. Gerontol. A Biol. Sci. Med. Sci.* **62,** 117–125.
151. Gispen, W.H. and Biessels, G.J. (2000) Cognition and synaptic plasticity in diabetes mellitus. *Trends Neurosci.* **23,** 542–549.
152. Mattson, M.P. (2003) Gene-diet interactions in brain aging and neurodegenerative disorders. *Ann. Intern. Med.* **139,** 441–444.
153. Prolla, T.A. and Mattson, M.P. (2001) Molecular mechanisms of brain aging and neurodegenerative disorders: lessons from dietary restriction. *Trends Neurosci*. **24,** S21–31.
154. Adams, M.M., Shi, L., Linville, M.C., Forbes, M.E., Long, A.B., Bennett, C., Newton, I.G., Carter, C.S., Sonntag, W.E., Riddle, D.R., and Brunso-Bechtold, J.K. (2008) Caloric restriction and age affect synaptic proteins in hippocampal CA3 and spatial learning ability. *Exp. Neurol.* **211,** 141–149.
155. Geng, Y.Q., Guan, J.T., Xu, M.Y., Xu, X.H., and Fu, Y.C. (2007) Behavioral study of calorie-restricted rats from early old age. *Conf. Proc. IEEE Eng. Med. Biol. Soc.* **2007**, 2393–2395.
156. Komatsu, T., Chiba, T., Yamaza, H., Yamashita, K., Shimada, A., Hoshiyama, Y., Henmi, T., Ohtani, H., Higami, Y., de Cabo, R., Ingram, D.K., and Shimokawa, I. (2008) Manipulation of caloric content but not diet composition, attenuates the deficit in learning and memory of senescence-accelerated mouse strain P8. *Exp. Gerontol.* **43,** 339–346.
157. Minor, R.K., Villarreal, J., McGraw, M., Percival, S.S., Ingram, D.K., and de Cabo, R. (2008) Calorie restriction alters physical performance but not cognition in two models of altered neuroendocrine signaling. *Behav. Brain Res.* **189,** 202–211.
158. Patel, N.V., Gordon, M.N., Connor, K.E., Good, R.A., Engelman, R.W., Mason, J., Morgan, D.G., Morgan, T.E., and Finch, C.E. (2005) Caloric restriction attenuates Abeta-deposition in Alzheimer transgenic models. *Neurobiol. Aging* **26,** 995–1000.
159. Wu, P., Shen, Q., Dong, S., Xu, Z., Tsien, J.Z., and Hu, Y. (2008) Calorie restriction ameliorates neurodegenerative phenotypes in forebrain-specific presenilin-1 and presenilin-2 double knockout mice. *Neurobiol. Aging* **29,** 1502–1511.
160. Raffaghello, L., Lee, C., Safdie, F.M., Wei, M., Madia, F., Bianchi, G., and Longo, V.D. (2008) Starvation-dependent differential stress resistance protects normal but not cancer cells against high-dose chemotherapy. *Proc. Natl. Acad. Sci. U. S. A.* **105,** 8215–8220.
161. Michishita, E., Park, J.Y., Burneskis, J.M., Barrett, J.C., and Horikawa, I. (2005) Evolutionarily conserved and nonconserved cellular localizations and functions of human SIRT proteins. *Mol. Biol. Cell* **16,** 4623–4635.

162. Ramadori, G., Lee, C.E., Bookout, A.L., Lee, S., Williams, K.W., Anderson, J., Elmquist, J.K., and Coppari, R. (2008) Brain SIRT1: anatomical distribution and regulation by energy availability. *J. Neurosci.* **28,** 9989–9996.
163. Li, Y., Xu, W., McBurney, M.W., and Longo, V.D. (2008) SirT1 inhibition reduces IGF-I/IRS-2/Ras/ERK1/2 signaling and protects neurons. *Cell Metab.* **8,** 38–48.
164. Boily, G., Seifert, E.L., Bevilacqua, L., He, X.H., Sabourin, G., Estey, C., Moffat, C., Crawford, S., Saliba, S., Jardine, K., Xuan, J., Evans, M., Harper, M.E., and McBurney, M.W. (2008) SirT1 regulates energy metabolism and response to caloric restriction in mice. *PLoS One* **3,** e1759.
165. Hisahara, S., Chiba, S., Matsumoto, H., Tanno, M., Yagi, H., Shimohama, S., Sato, M., and Horio, Y. (2008) Histone deacetylase SIRT1 modulates neuronal differentiation by its nuclear translocation. *Proc. Natl. Acad. Sci. U. S. A.* **105,** 15599–15604.
166. Tang, B.L. and Chua, C.E. (2008) SIRT1 and neuronal diseases. *Mol. Aspects Med.* **29,** 187–200.
167. Alcain, F.J. and Villalba, J.M. (2009) Sirtuin activators. *Expert Opin. Ther. Pat.* **19,** 403–414.
168. Araki, T., Sasaki, Y., and Milbrandt, J. (2004) Increased nuclear NAD biosynthesis and SIRT1 activation prevent axonal degeneration. *Science* **305,** 1010–1013.
169. Wang, J., Zhai, Q., Chen, Y., Lin, E., Gu, W., McBurney, M.W., and He, Z. (2005) A local mechanism mediates NAD-dependent protection of axon degeneration. *J. Cell Biol.* **170,** 349–355.
170. Chen, J., Zhou, Y., Mueller-Steiner, S., Chen, L.F., Kwon, H., Yi, S., Mucke, L., and Gan, L. (2005) SIRT1 protects against microglia-dependent amyloid-beta toxicity through inhibiting NF-kappaB signaling. *J. Biol. Chem.* **280,** 40364–40374.
171. Julien, C., Tremblay, C., Emond, V., Lebbadi, M., Salem, N., Jr., Bennett, D.A., and Calon, F. (2009) Sirtuin 1 reduction parallels the accumulation of tau in Alzheimer disease. *J. Neuropathol. Exp. Neurol.* **68,** 48–58.
172. Qin, W., Zhao, W., Ho, L., Wang, J., Walsh, K., Gandy, S., and Pasinetti, G.M. (2008) Regulation of forkhead transcription factor FoxO3a contributes to calorie restriction-induced prevention of Alzheimer's disease-type amyloid neuropathology and spatial memory deterioration. *Ann. N. Y. Acad. Sci.* **1147,** 335–347.
173. Tang, B.L. (2009) Sirt1's complex roles in neuroprotection. *Cell. Mol. Neurobiol.* **29(8),** 1093–1103.
174. Alcain, F.J. and Villalba, J.M. (2009) Sirtuin inhibitors. *Expert Opin. Ther. Pat.* **19,** 283–294.
175. Chong, Z.Z., Lin, S.H., Li, F., and Maiese, K. (2005) The sirtuin inhibitor nicotinamide enhances neuronal cell survival during acute anoxic injury through AKT, BAD, PARP, and mitochondrial associated "anti-apoptotic" pathways. *Curr. Neurovasc. Res.* **2,** 271–285.
176. Han, M.K., Song, E.K., Guo, Y., Ou, X., Mantel, C., and Broxmeyer, H.E. (2008) SIRT1 regulates apoptosis and Nanog expression in mouse embryonic stem cells by controlling p53 subcellular localization. *Cell Stem Cell* **2,** 241–251.
177. Kakefuda, K., Fujita, Y., Oyagi, A., Hyakkoku, K., Kojima, T., Umemura, K., Tsuruma, K., Shimazawa, M., Ito, M., Nozawa, Y., and Hara, H. (2009) Sirtuin 1 overexpression mice show a reference memory deficit, but not neuroprotection. *Biochem. Biophys. Res. Commun.* **387,** 784–788.
178. Bekinschtein, P., Katche, C., Slipczuk, L.N., Igaz, L.M., Cammarota, M., Izquierdo, I., and Medina, J.H. (2007) mTOR signaling in the hippocampus is necessary for memory formation. *Neurobiol. Learn. Mem.* **87,** 303–307.
179. Slipczuk, L., Bekinschtein, P., Katche, C., Cammarota, M., Izquierdo, I., and Medina, J.H. (2009) BDNF activates mTOR to regulate GluR1 expression required for memory formation. *PLoS One* **4,** e6007.
180. Berger, Z., Ravikumar, B., Menzies, F.M., Oroz, L.G., Underwood, B.R., Pangalos, M.N., Schmitt, I., Wullner, U., Evert, B.O., O'Kane, C.J., and Rubinsztein, D.C. (2006) Rapamycin alleviates toxicity of different aggregate-prone proteins. *Hum. Mol. Genet.* **15,** 433–442.
181. Ravikumar, B., Vacher, C., Berger, Z., Davies, J.E., Luo, S., Oroz, L.G., Scaravilli, F., Easton, D.F., Duden, R., O'Kane, C.J., and Rubinsztein, D.C. (2004) Inhibition of mTOR induces autophagy and reduces toxicity of polyglutamine expansions in fly and mouse models of Huntington disease. *Nat. Genet.* **36,** 585–595.
182. Goorden, S.M., van Woerden, G.M., van der Weerd, L., Cheadle, J.P., and Elgersma, Y. (2007) Cognitive deficits in Tsc1+/- mice in the absence of cerebral lesions and seizures. *Ann. Neurol.* **62,** 648–655.
183. Chen, Q., Ding, Q., and Keller, J.N. (2005) The stationary phase model of aging in yeast for the study of oxidative stress and age-related neurodegeneration. *Biogerontology* **6,** 1–13.
184. Gancedo, J.M. (2008) The early steps of glucose signalling in yeast. *FEMS Microbiol. Rev.* **32,** 673–704
185. Birkenkamp, K.U. and Coffer, P.J. (2003) Regulation of cell survival and proliferation by the FOXO (Forkhead box, class O) subfamily of Forkhead transcription factors. *Biochem. Soc. Trans.* **31,** 292–297.
